# *De novo* Lesions Frequently Develop in Adult Normal Thyroid Over Almost Six Years

**DOI:** 10.3389/fendo.2020.00018

**Published:** 2020-01-29

**Authors:** Giulia Brigante, Maria Laura Monzani, Michela Locaso, Valentina Luisa Gnarini, Luigi Graziadei, Shaniko Kaleci, Maria Cristina De Santis, Simonetta Tagliavini, Manuela Simoni, Vincenzo Rochira, Bruno Madeo

**Affiliations:** ^1^Unit of Endocrinology, Department of Biomedical, Metabolic and Neural Sciences, University of Modena and Reggio Emilia, Modena, Italy; ^2^Department of Medical Specialties, Azienda Ospedaliero-Universitaria di Modena, Modena, Italy; ^3^Department of Diagnostic Medicine, Clinics and Public Health, Azienda Ospedaliero-Universitaria di Modena, Modena, Italy; ^4^Department of Laboratory Medicine and Pathology, Azienda Ospedaliero-Universitaria di Modena, Modena, Italy

**Keywords:** healthy thyroid, thyroid nodules, follow-up, ultrasound, incidence

## Abstract

**Purpose:** In order to understand how thyroid abnormalities emerge over time in adults, we evaluated incidence of thyroid diseases in healthy subjects, after almost 6 years from a previous negative ultrasound.

**Methods:** Anamnestic and physical data were collected. Ultrasound neck evaluation was performed by an experienced endocrinologist, recording detailed thyroid and nodules characteristics. Nodules were classified according to American Thyroid Association classification for prediction of cancer risk. Serum samples were collected for subsequent evaluations (TSH, free thyroid hormones, calcitonin, anti-thyroid antibodies). Anamnestic, clinical, sonographic, and serological characteristics were analyzed with logistic regression analysis for subjects with nodules vs. those without.

**Results:** One hundred and eleven subjects were enrolled (43M, 68F). Half of them developed nodules, mainly smaller than 1 cm and without suspicious characteristics. Ninety-seven percent were euthyroid. Only 4% had serological diagnosis of thyroiditis. Incidence of thyroid diseases was higher in women, especially nulliparous. Comparing clinical characteristics of subjects with and without nodules, the only statistically significant difference concerned thyroid volume adjusted for body weight or surface (*p* < 0.05), but not residual volume excluding nodules. Multivariate logistic regression analysis showed that female gender, higher BMI-adjusted thyroid volume and residual thyroid volume excluding nodules, nulliparity, age, and fT3 increase the risk of developing nodules.

**Conclusions:** These results demonstrate that adult thyroid tissue undergoes changes that are already detectable by US after almost 6 years. Half of the enrolled subjects developed *de novo* nodules or colloid cysts of poor clinical relevance.

## Introduction

Thyroid nodules represent a common clinical finding and their prevalence is increasing worldwide (1). Clinically silent thyroid lesions are incidentally detected at ultrasound (US) in 20–76% of the general population (2–4), with prevalence rates similar to those reported by autopsy studies (5). However, screening for thyroid cancer in asymptomatic adults is not recommended (1, 6), considering its rarity and predominantly favorable prognosis. Moreover, there is no observational evidence of changes in mortality after introduction of a mass screening program.

In a previous study, we found a high prevalence of US thyroid abnormalities in volunteers unaware of any thyroid disease: up to 51% of subjects presented some US abnormalities, including nodular goiter (45%) and thyroiditis (6%) (7). They were treated according to clinical practice, revealing a 2% frequency of malignant lesions. The remaining half, with normal thyroid US, was discharged and no further investigations were indicated, according to guidelines (8).

Recent data question the need for close follow-up in subjects with asymptomatic, sonographically or cytologically benign thyroid nodules (9), reassuring on the benignity of the vast majority of thyroid nodules. However, the incidence of thyroid nodules in adults with previously documented negative US is unknown.

A study of the early ‘90s demonstrated an increasing incidence of thyroid abnormalities in a cohort of subjects tested with a 20 year interval between the first and the second evaluation (10). In the initial examination, 3.7% of subjects aged between 11 and 18 years presented thyroid abnormalities. Twenty years later, incidence grew up to 10%. However, subjects were only screened by physical examination and it is well-known that nearly half of nodules detected by US, even >1 cm, escape the physical evaluation (11). Moreover, the young age of subjects at the first evaluation explains such low incidence of thyroid diseases.

To our knowledge, there are no data about the possible occurrence of nodules in adults with previous documented absence of US thyroid abnormalities. The aim of this study was to evaluate incidence of thyroid disease in subjects with previously negative thyroid US. We also evaluated any patient characteristic able to predict the risk of developing thyroid nodules, the incidence of palpable thyroid nodules at physical examination, thyroiditis, and pituitary-thyroid axis function changes.

## Materials and Methods

### Study Design and Subjects

Subjects enrolled in this prospective study were selected among those whit normal thyroid US at least 5 years before. One hundred subjects had already been enrolled as healthy volunteers for a previous study (7). They had been recruited by local advertisement as the control group for an ongoing case-control study on thyroid cancer. Other 36 subjects were recruited from the participants in another ongoing case-control study on thyroid cancer, with the same inclusion/exclusion criteria and the same methodological approach. Among the 136 subjects evaluated at baseline, 111 agreed to undergo a second evaluation. The first evaluation, published in Gnarini et al. (7), was performed between 2011 and 2013. The second one between 2017 and 2018. All the participants underwent clinical examination and neck US scan. Subjects were also asked to undergo a blood test to collect serum samples.

### Anamnesis and Physical Examination

Anamnesis was mainly focused on family history of thyroid disease, previous exposure to neck-mediastinum radiation therapy, previous pregnancies, current or previous smoking habit, onset of pain or compression in the anterior neck, drug history, access to endocrinological department for an in-between neck US scan or thyroid function evaluation.

During physical examination, we checked thyroid morphology at palpation, anthropometric measurements and blood systolic and diastolic pressure [mmHg]. At thyroid palpation, the following items were collected: gland palpability, volume, consistency, surface, mobility on swallowing, and achiness; thyroiditis pattern; presence of palpable nodules; nodule position, dimensions, consistency, mobility on swallowing and achiness.

Anthropometric measurements included patient's weight, height and body mass index (BMI). Height [m] and weight [kg] were measured using standard anthropometric techniques. BMI was calculated as the ratio of weight in kg to the square of height in meters (m). Body surface area (BSA) (m^2^) was calculated using Mosteller method: [height (m) x weight (kg)/36]^½^.

### US Scan

US scan was performed with a Siemens Acuson Antares^®^ (Philadelphia, USA, 10 MegaHertz-linear scanner, B mode), the same used in the previous evaluation (7), by a single expert endocrinologist with 15 years of thyroid US experience (MB), assisted by an operator in training (GVL or BG). The same operators performed baseline and second evaluation.

The following data regarding thyroid gland were reported: three-dimension diameters of thyroid lobes [sagittal (AP), transversal (T) and longitudinal (L)], the presence of nodules and/or thyroiditis according to standard criteria (12).

Detected nodules were described according to dimensions (AP, T and L), localization in the gland, shape, composition (liquid, solid, mixed), echogenicity (anechoic, hypoechoic, isoechoic, hyperechoic), margins (regular, irregular), presence or absence of microcalcifications, vascularization by echo-color-doppler. Then, nodules were evaluated according to clinical practice and international guidelines (13). Each nodule was classified according to American Thyroid Association (ATA) classification for prediction of thyroid cancer risk: ATA1, benign, purely cystic nodule; ATA2, very low suspicion, spongiform or partially cystic nodule without any of the US features described in low-, intermediate- or high-suspicion patterns; ATA3, low suspicion, isoechoic or hyperechoic solid nodule, or partially cystic nodule with eccentric solid area without microcalcifications, irregular margins, extrathyroidal extension, taller than wide shape; ATA4, intermediate suspicion, hypoechoic solid nodule with smooth margins without microcalcifications, extrathyroidal extension or taller than wide shape; ATA5, high suspicion, solid hypoechoic nodule or solid hypoechoic component of partially cystic nodule with one or more of irregular margins (infiltrative, microlobulated), microcalcifications, taller than wide shape, rim calcifications with small extrusive soft tissue component, evidence of extrathyroidal extension (13).

According to ATA guidelines, nodules above 10 mm presenting US characteristics suspicious for malignancy or uncertain to be intra or extra-thyroidal, were referred to US-guided fine needle aspiration (FNA) and the cytological result guided subsequent therapeutic choices.

Sonographic thyroiditis was defined in case of diffusely heterogeneous gland appearance with a marked diffuse reduction of the gland echogenicity together with admixed hypo- and hyperechoic non-nodular areas (14). When clinical, sonographic and/or biochemical thyroiditis was diagnosed, subjects were treated accordingly.

Thyroid volume (TV) was estimated by summing the volume of the two lobes obtained in accordance with the formula [length (cm) x width (cm) x depth (cm) x correction factor 0.479] (15). In addition, TV was normalized by correcting for the anthropometric parameters BSA and BMI.

### US-Guided FNA and Cytological Analysis

According to international guidelines (13), selected nodules underwent US guided FNA. After obtaining patient consent, aspiration was performed using a 22–23 gauge needle under US assistance. Both US and FNA were performed by the same investigator (MB) in all subjects. The aspirates were fixed in alcohol and stained with hematoxylin and eosin. Then, cytological specimens were classified by expert pathologists, according to the Italian consensus for the classification and reporting of thyroid cytology (16).

### Serum Measurements

Serum samples were analyzed for evaluating pituitary-thyroid axis function, measuring thyroid stimulating hormone (TSH), free thyroxine (fT4), and free triiodothyronine (fT3) by enzyme immunoassays with chemiluminescent microparticle technology (Abbott Architect Analyzer; TSH: intra-assay coefficient of variation (CV) 3.10% and inter-assay CV 3.50%, normal range: 0.35–4.94 μIU/mL; fT4: intra-assay CV 3.80% and inter-assay CV 5.70%, normal range: 7–15 pg/mL; fT3 intra-assay CV 2.80% and inter-assay CV 3.65%, normal range: 1.7–3.7 pg/mL). Moreover, anti-thyroperoxidase antibodies (TPOAb), anti-thyroglobulin antibodies (TgAb), and calcitonin were measured in all subjects. Chemiluminescence immunoassays were employed for TgAb (Access, Beckmann Coulter, United States; intra-assay CV 4.55% and inter-assay CV 2.80%, normal range: 0–4 UI/mL) and TPOAb (Access, Beckmann Coulter, Ireland; intra and inter-assay CV 5.80%, normal range: 0–9 UI/mL) assays. Calcitonin was measured by ultrasensitive chemiluminescence immunoassay (Liason CT, DiaSorin, Vercelli, Italy; intra and inter-assay CV 6.0% normal range: 0–12 pg/mL in males, 0–7.5 pg/mL in females).

### Ethics Statement

All procedures performed in studies involving human participants were in accordance with the ethical standards of the institutional and/or national research committee (Comitato Etico di Modena, number 21/17) and with the 1,964 Helsinki declaration and its later amendments or comparable ethical standards. Informed consent was obtained from all individual participants included in the study.

### Statistical Analysis

Statistical analysis was performed using STATA^®^ software version 14 (StataCorp. 2015. Stata Statistical Software: Release 14. College Station, TX: StataCorp LP.). Descriptive statistics were presented for baseline demographic clinical characteristics for the entire group, as well as for the groups of patients with and without nodules.

Continuous variables were presented as the number of patients (N), mean, standard deviation (SD), and compared between subgroups using Unpaired Student's *t* test; while categorical variables were presented as frequency (N, percentage [%]) and compared using Pearson's chi-squared test.

A multivariate logistic regression model was carried out using a stepwise selection method to identify the prognostic factors for the occurrence of nodules. In the first step, the intercept-only model was fitted and individual score statistics for the potential variables were evaluated. A significance level of 0.05 was used to allow a variable into the model. In stepwise selection, an attempt was made to remove any insignificant variables from the model before adding a significant variable to the model. Hosmer and Lemeshow test was used to evaluate “goodness of fit” in the selection model. Data from the multivariate logistic regression analyses were expressed as odds ratio (OR) and 95% confidence interval (CI). A *P* < 0.05 was considered statistically significant.

## Results

One hundred and eleven subjects were enrolled, with a mean interval between the first and the second thyroid evaluation of 6 ± 0.56 years (min 5, max 7). The mean age was 51 ± 12 years (min 27, max 78). Subject characteristics are summarized in Table 1.

**Table 1:** Population characteristics.

**TOTAL SUBJECTS**	**111**
Sex (n)	
M	43 (39%)
F	68 (61%)
Age (years)	51 ± 12
Time from first thyroid evaluation (years)	6 ± 0.56
Smoking habit (n)	
No	71 (64%)
Ex	27 (24%)
Yes	13 (12%)
Positive family history (n)	
For thyroid benign alterations	43 (39%)
For thyroid malignant nodules	7 (6%)
No	61 (55%)
BMI (kg/m^2^)	26.3 ± 5.7
No. of patients with nodules	54 (49%)
No. of nodules	106
Thyroiditis	
Biochemical diagnosis alone^*^	3 (3%)
US diagnosis alone°	3 (3%)
Both biochemical and US diagnosis	1 (1%)
Thyroid function	
Euthyroidism	108 (97%)
Subclinical hyperthyroidism	2 (2%)
LT4 treated hypothyroidism	1 (1%)

Values are expressed as total and percentage (%) or mean and standard deviation [BMI, body mass index; US, ultrasound; LT4, levothyroxine; ^*^high anti-thyroperoxidase antibodies and/or anti-thyroglobulin antibodies; °US features consistent with thyroiditis].

No subjects were exposed to high-dose ionizing radiation for therapeutic purposes.

Fifteen subjects reported to have undergone thyroid US or TSH measurement elsewhere, between first and second evaluation in our center. Seven subjects underwent neck US because of extra-thyroid abnormalities (salivary gland study or lymphadenopathy) or for screening program at work. Eight subjects measured TSH levels on the advice of the general physician in routine blood tests. They all were asymptomatic when such evaluations were performed. Among them, two were diagnosed with nodular lesions and one with hypothyroidism treated with levothyroxine.

No nodules were detected upon thyroid palpation.

Thanks to US, 106 nodules were diagnosed in 54 (49%) subjects. Half of them had a single nodule; 14 (26%) had 2 nodules; 8 (15%) had 3 nodules; 2 (3.7%) had 4 nodules; 1 (1.8%) patient had 5 nodules; 1 (1.8%) had 6 nodules; 1 (1.8%) had 8 nodules.

Considering gender, nodules developed in 18 males (42% of total men) and 36 females (53% of total women). Accordingly, 2 out of 3 subjects with nodules were women (66.7%).

Among females, nulliparous resulted at higher risk: 70% of them developed nodules, while only 47% of women with at least one previous pregnancy had nodular lesions.

Mean dimensions of nodules were 2.3 ± 1.4 mm (AP), 2.9 ± 1.8 mm (T), and 3.6 ± 2.4 mm (L). Only three nodules had a larger diameter above 10 mm.

Nodules were classified according to their US characteristics, considering ATA reporting system (Table 2).

**Table 2:** Nodules distribution according to the American Thyroid Association classification and nodule dimension (larger diameter was considered) ATA1, benign, purely cystic nodule; ATA2, very low suspicion; ATA3, low suspicion; ATA4, intermediate suspicion; ATA5, high suspicion.

	**Nodule larger diameter**		
	** < 5 mm** **(*n* = 93)**	***5-10 mm*** ***(n = 10)***	**> 10 mm** (*n***= 3)**
ATA1 (*n* = 66)	64 (69%)	2 (20%)	0
ATA 2 (*n* = 11)	8 (8%)	1 (10%)	2^**x**^ (67%)
ATA 3 (*n* = 15)	9 (10%)	5 (50%)	1^*^ (33%)
ATA 4 (*n* = 11)	10 (11%)	1 (10%)	0
ATA 5 (*n* = 3)	2 (2%)	1 (10%)	0

Percentages are referred to the total number of nodules with the same diameter; **x** one of these nodules was subjected to fine needle aspiration because of uncertain intra- or extra-thyroidal position (final citology: non-diagnostic, with high parathormone levels on washing liquid, suggestive for hypertrophic parathyroid); ^*^subjected to fine needle- aspiration (final citology: low-risk indeterminate lesion).

Nodule position was evaluated: 5 were in isthmus, 59 in the right lobe (16 inferior, 24 median, 19 superior), 41 in the left lobe (16 inferior, 13 median, 12 superior). Most suspicious nodules were located in the upper pole. In particular, 20% of nodules in the upper pole were classified as ATA 4 or ATA 5, against the 11% in the middle portion and 9% in the lower portion.

Most nodules were liquid (63%), followed by solid (25%), mixed (10%), and spongiform (2%). Regarding echogenicity, 63% of nodules were anechoic, 15% isoechoic, 13% hypoechoic, 6% iso-hypoechoic, 2% hyperechoic, 1% markedly hypoechoic. Only one nodule had an incomplete halo, the 14% had a complete halo and the remaining 85% were not haloed. Most nodules (68%) were not vascularized. Twenty seven percent of nodules had a peripheral vascularization, 4% had also intra-nodular vascularization, and hilar vascularization was detected in the nodule that then proved to be a parathyroid.

Two subjects (4% of subjects presenting nodules) underwent FNA. One was performed because of uncertain intra- or extra-thyroidal position. The cytological result was not diagnostic (Tir 1) with high parathormone levels on washing liquid, suggestive for hypertrophic parathyroid. Consequently, this lesion was excluded from subsequent analysis. The second FNA was prudentially performed on an ATA3 nodule with a maximum diameter of 13 mm (at the limit with respect to the dimensional cutoff given by the ATA guidelines). Cytology resulted compatible with low-risk indeterminate lesion (Tir 3A). The patient was recommended to continue US follow-up. The nodule maintained same dimensions and characteristics over time. Patient is still euthyroid and serum calcitonin levels were normal.

US pattern consistent with thyroiditis was present in 4 (3.6%) subjects. Other 4 subjects (3.6%) had US findings of both nodules and thyroiditis.

Mean TSH level was 1.41 ± 0.74 μIU/mL. TSH was below normal in 2 (1.8%) subjects, with values of 0.26 and 0.03 μIU/mL, respectively, with fT3 and fT4 within normal ranges. Average fT4 and fT3 levels were 10.50 ± 1.33 pg/mL and 2.66 ± 0.33 pg/mL, respectively. With regard to anti-thyroid antibodies, one (0.9%) patient had TgAb above the normal level; two (1.8%) had high TPOAb level, and 1 (0.9%) had high levels of both TgAb and TPOAb. Consequently, 4 cases (3.6%) of bio-humoral thyroiditis were identified; only 1 of them had US features consistent with thyroiditis. Two-third of the subjects with US and/or serological thyroiditis were females. Mean calcitonin level was 1.40 ± 2.65 pg/mL. Two subjects had calcitonin levels slightly above normal (1 male with 14.6 pg/mL and 1 female with 7.7 pg/mL), not clinically relevant (17).

Anamnestic data, clinical and sonography findings, and biochemical analyses of subjects with and without nodules were compared (Table 3). Thyroid volume, after correction for BSA or BMI, was significantly higher in subjects with nodules. Excluding volume of nodules, the surrounding thyroid was not significantly larger in subjects who developed nodules compared to those who did not (p = 0.12). Apart from thyroid volume, no other clinical data differed between subjects with and without nodules (Table 3). Serum analyses were not correlated with nodular disease, ruling out a direct link between nodules incidence and TSH (*p* = 0.23), fT4 (*p* = 0.59), fT3 (*p* = 0.43), TgAb (*p* = 0.27), TPOAb (*p* = 0.74).

**Table 3:** Comparison between subjects with and without thyroid nodules.

	**Nodules** **YES (*n* = 54)**	**Nodules NO** **(*n* = 57)**	***p*-value**
Sex			
M	18 (33.3)	25 (43.9)	0.255
F	36 (66.7)	32 (56.1)	
Time from first thyroid evaluation (years)	6.1 ± 0.5	5.9 ± 0.6	0.100
Age (years)	51.1 ± 11.1	49.8 ± 12.5	0.566
Family history (n)			
For thyroid benign alterations	25 (46%)	18 (32%)	0.282
For thyroid malignancy	3 (6%)	4 (7%)	
Absent	26 (48%)	35 (61%)	
Weight (Kg)	69.7 ± 15.6	73.4 ± 16.8	0.233
BMI (Kg/m^2^)	25.4 ± 4.8	27.1 ± 6.3	0.109
Systolic blood pressure (mmHg)	115.4 ± 17.1	116.3 ± 14.1	0.774
Diastolic blood pressure (mmHg)	71.8 ± 10.8	72.0 ± 9.3	0.931
Smoking habit (n)			
No	34 (63%)	37 (65%)	0.923
Ex	7 (13%)	6 (10%)	
Yes	13 (24%)	14 (25%)	
Thyroid volume (mL)	8.2 ± 2.8	7.3 ± 2.1	0.108
BSA-adjusted thyroid volume (mL/m^2^)	4.6 ± 1.4	4.0 ± 1.2	*0.042*
BMI-adjusted thyroid volume (mL/Kg/m^2^)	0.33 ± 0.12	0.28 ± 0.9	*0.048*
Residual thyroid volume (mL)	7.87 ± 0.8	7.3 ± 2.1	0.122
TSH (μIU/mL)	1.31 ± 0.75	1.48 ± 0.71	0.229
fT4 (pg/mL)	10.6 ± 1.29	10.4 ± 1.37	0.594
fT3 (pg/mL)	2.7 ± 0.28	2.6 ± 0.36	0.429
TgAb (IU/mL)	0.2 ± 0.72	1.6 ± 8.82	0.268
TPOAb (IU/mL)	2.4 ± 11.75	3.3 ± 16.86	0.748

*Values are expressed as total and percentage (%) or mean and standard deviation (SD). P-value < 0.05 (italics) was considered statistically significant (statistical differences were examined using Student's t test for continuous variable, chi-square and Fisher's exact test for categorical variable) [BMI, body mass index; BSA, body surface area; Residual thyroid volume, thyroid volume excluding nodules; TSH, thyroid stimulating hormone; fT4, free thyroxyne; fT3, free triiodothyronine; TgAb, anti-thyroglobulin antibodies; TPOAb, anti-thyroperoxidase antibodies]*.

Finally, logistic regression analysis was performed, considering all collected data. Upon univariate, logistic regression, most of the anamnestic, physical, sonographic, or serological values were not predictive of the risk of developing thyroid nodules, except BMI-adjusted thyroid volume (*p* = 0.025). When considered together, at the multivariate logistic regression, more variables proved to be significant (Table 4). Females had 27.53 times greater relative odds for presence of nodules, compared to males (*p* < 0.001). This significance still remains even removing parity, which could represent a bias. Moreover, for an increase of 1 mL/Kg/m^2^ of BMI-adjusted volume, nodule risk was 848-fold increased (*p* = 0.002). Also increased residual thyroid volume, excluding nodules, resulted a risk factor for nodules (*p* = 0.016). Previous pregnancy resulted to be a protective factor (*p* = 0.004). Viceversa, higher fT3 levels and age were identified as predictors of new nodules (*p* = 0.018 and 0.047, respectively).

**Table 4:** Multivariate logistic regression model comparing subjects with and without thyroid nodules.

**Variable**	**Multivariate OR** **(95%CI)**	***p*-value**
Gender, F	27.53 (4.72–160.40)	<0.001
Age	1.04 (1.01–1.08)	0.047
BMI-adjusted-TV	848.03(11.27–63763.62)	0.002
Residual TV	1.41 (1.06–1.86)	0.016
Previous pregnancy	0.10 (0.02–0.48)	0.004
FT3	7.12 (1.40–36.25)	0.018

*Only significant variables are shown (OR, odds ratio; CI, confidence interval; BMI, body mass index; TV, thyroid volume; Residual TV, thyroid volume excluding nodules; fT3, free triiodothyronine)*.

## Discussion

This prospective study demonstrates that new thyroid lesions may occur in adult subjects without previous US abnormalities. Former data showed that 9% of subjects affected by nodular goiter should develop new nodules in 5 years (9). Albeit with the limit of small sample size, our results suggest the possible occurrence of lesions even in healthy thyroids in a time frame of about 6 years. However, the vast majority (65%) of *de novo* lesions was purely cystic or spongiform, therefore of little clinical significance.

Both the high intrinsic growth potential of thyrocytes and the hetereogeneity of thyroid growth are well-known and can provide a plausible explanation of nodular trasformation of thyroid tissue (18). The present study demonstrates that thyroid volume increases as a result of nodule growth, only in correspondence of lesions, without affecting the surrounding tissue. We confirmed that the growth of thyroid nodules is a local process, not associated with growth of the paranodular tissue (19).

Considering gender, our results confirm the well-known greater prevalence of thyroid diseases (including both nodules and thyroiditis) in women compared to men (3). Females have higher incidence of both nodules and thyroiditis, compared to males. Female gender is a significant risk factor when considered together with age, BMI-adjusted thyroid volume, residual thyroid volume excluding nodules, previous pregnancies, and thyroid function at multivariate regression analysis. Interestingly, nulliparous females have an even higher risk. Previous pregnancies are known to be associated with slower nodule growth too (9). Thus, male gender and previous pregnancies seem to be somehow protective from nodule occurrence and evolution. This is in contrast with studies reporting an increased prevalence of thyroid nodules in women with higher gravidity (20). Considering the increased iodine requirement during pregnancy and its effect on nodular growth, we cannot draw definitive conclusion on this topic as we do not have information about iodine status in our cohort.

Together with US appearance, we evaluated thyroid functionality. TSH and free thyroid hormone levels did not differ between subjects who developed nodules or not, suggesting pathophysiological independence of nodular lesions from thyroid function. Surprisingly, higher fT3 levels were identified as related to the appearance of new nodules, but only when considered together with other factors. Since fT3 reliability is known to be limited (21), we believe that this statistical significance must be verified on a larger sample and with more reliable dosage methods before any physio-pathological hypotheses can be made.

Again, no significant differences were found regarding age, physical examination, family history for thyroid diseases, smoking habit, thyroid function or Ab levels comparing subjects developing or not developing nodules in the observation period. Only thyroid volume adjusted for body surface or BMI resulted significantly increased in subjects with nodules, maybe as a consequence of nodules appearance. In fact, when residual thyroid volume excluding the cumulative nodule volume was considered, no significant difference was found between subjects with nodules compared to others. However, multivariate logistic regression analysis showed that residual thyroid volume, together with female gender, higher BMI-adjusted thyroid volume, nulliparity, age and fT3 increases risk of developing thyroid nodules.

The practical clinical implication of these results is that re-evaluation of subjects with previously negative thyroid US is not justified, at least within a 6 year time frame. Although half of the subjects developed nodules, homogeneously divided between single and multiple, most of them had no dimensional or sonographic clinically relevant features. The great majority of lesions was under 10 mm, without suspicious US appearance. Only 14 nodules were assigned to higher risk ATA classes; they all were smaller than 1 cm, and 12 were even below 5 mm. According to the very small dimensions, no nodules were detectable at neck palpation even when lesions were found at the following US. Thus, we confirm that physical examination cannot be considered a first-line approach to detect any thyroid nodule, even if conducted by an expert clinician (11).

Obviously, only histological confirmation could endorse the benign nature of the lesions we found. However, US characteristics and the codification in the ATA classification allowed us to exclude with a high probability the presence of suspicious nodules. And it is well-known that most thyroid nodules are benign and do not progress to malignancy (9).

The results of the present study could be limited by lack of validation by an external operator and small sample size. Therefore, validation studies on larger series are needed to confirm the presented data and clarify any possible criteria to define if and which healthy subjects need to repeat thyroid US, in the absence of clinical suspicion. Moreover, we do not have information about iodine status in the enrolled subjects, thus losing the role of an important goitrogenic factor.

In conclusion, these results provide new insights on the natural history and pathophysiology of thyroid, suggesting that *de novo* lesions may develop in adult normal thyroid in a time frame of about 6 years, generating nodules detectable at standard US evaluation. From a clinical point of view, the small dimension and the poor clinical relevance of new nodules does not justify US repetition after only 6 years in subjects with a previous negative neck scan.

## Data Availability Statement

The datasets generated for this study are available on request to the corresponding author.

## Author Contributions

Material preparation, data collection were performed by GB, MM, ML, LG, VG, BM, VR, MS, and analysis were performed by GB, SK, MD, ST, and BM. The first draft of the manuscript was written by GB. All authors commented on previous versions of the manuscript, read and approved the final manuscript, contributed to the study conception and design.

### Conflict of Interest

The authors declare that the research was conducted in the absence of any commercial or financial relationships that could be construed as a potential conflict of interest.
